# Vitamin C deficiency in a Northern Saskatchewan Population, Canada

**DOI:** 10.1371/journal.pgph.0007464

**Published:** 2026-10-28

**Authors:** Jeffrey David Connor Irvine, Yoseph C. Atreyu, Mary Carlson, Brian Quinn, James Irvine, Nnamdi Ndubuka

**Affiliations:** 1 Northern Medical Services, Department of Family Medicine, College of Medicine, University of Saskatchewan, Saskatchewan, Canada; 2 Lac La Ronge Indian Band Health Services, La Ronge, Saskatchewan, Canada; 3 Population Health Unit, Saskatchewan Health Authority, Saskatchewan, Canada; 4 Department of Family Medicine, College of Medicine, University of Saskatchewan, Saskatchewan, Canada; 5 Northern Inter-Tribal Health Authority, Prince Albert, Saskatchewan, Canada; 6 Community Health and Epidemiology, College of Medicine, University of Saskatchewan, Saskatchewan, Canada; PLOS: Public Library of Science, UNITED STATES OF AMERICA

## Abstract

Vitamin C deficiency, shaped by systemic inequities and disrupted food systems, remains a concern in remote Indigenous communities. In Northern Saskatchewan, clinicians observed increasing suspected scurvy among adults from geographically isolated First Nations communities. We conducted an exploratory retrospective review of adults who underwent clinician-initiated serum ascorbic acid testing at the La Ronge Medical Clinic from January 2011 to March 2024. Testing was based on clinical suspicion or perceived risk rather than population-based screening. Vitamin C deficiency was defined as <11 µmol/L and hypovitaminosis C as 11–23 µmol/L. Demographic, clinical, and testing-indication data were extracted, with exploratory comparisons by First Nations status, sex, age, and documented characteristic scurvy features. Study governance followed OCAP principles through partnerships with Lac La Ronge Indian Band Health Services and the Northern Inter-Tribal Health Authority. Among 48 individuals with analyzable results, 27 (56%) had vitamin C deficiency and 10 (21%) had hypovitaminosis C. Deficiency was more common among First Nations than non-First Nations patients (66% versus 20%, p = 0.014). Dermatologic and oral manifestations were common indications for testing, although deficiency was also identified among patients without documented characteristic scurvy features. The high proportion of deficiency among tested patients suggests that vitamin C deficiency is an important clinical concern in this setting. However, this clinically selected sample should not be interpreted as estimating population-level prevalence. Given restricted laboratory access and the technical challenges of serum ascorbic acid testing, empiric supplementation may be a practical option for high-risk individuals. Longer-term responses should prioritize Indigenous-led food sovereignty, culturally grounded interventions, and policies addressing food access and underlying structural inequities.

## Introduction

Scurvy, or clinical vitamin C deficiency, once confined to historical contexts of malnutrition and deprivation, has re-emerged as a global health equity issue, including within high-income countries [1–3]. Vitamin C (ascorbic acid) is a water-soluble vitamin essential for collagen synthesis, immune function, and antioxidant activity [4]. Not produced by the human body, ascorbic acid must be consumed through foods such as fresh fruits and vegetables and some organ meats [5], although it degrades with prolonged shelf life and excessive cooking [4]. Deficiency symptoms of impaired collagen synthesis can arise within weeks of severely limited intake, leading to capillary fragility, microcytic anemia, poor wound healing, and gingival hypertrophy [6]. Fatigue, mood change, or joint pain may indicate subclinical deficiency [6] and may be misattributed in modern clinical practice. If untreated, scurvy can be life-threatening [3]. Recent global reviews underscore that even marginal deficiencies can contribute to increased oxidative stress and impaired immune responses, potentially exacerbating chronic diseases in vulnerable populations [3].

Scurvy was first reported in Canada in 1535, when members of Jacques Cartier’s expedition wintering near Quebec City developed the disease [7]. Many people died until the local Haudenosaunee people shared their traditional remedy, possibly eastern white cedar or balsam fir, which successfully treated the sailors [7]. Accounts from the Parry Arctic expedition describe sprouting mustard and cress in ship cabins as successful antiscorbutic remedies [8], a practice still available in modern household microgreen cultivation.

In recent years, anecdotal reports from local clinicians raised suspicions of scurvy [9]. This was concerning given the dietary shifts away from nutrient-dense, plant-based foods and fresh organ meats towards packaged, micronutrient-poor ultra-processed foods [10]. The family physicians at the local medical clinic in La Ronge, a relatively-remote community in northern Saskatchewan, Canada, and the nurses at the surrounding outpost stations serve a region with a population of approximately 16,000, of whom 83.9% identify as Indigenous and 75.2% as First Nations according to the 2021 Statistics Canada Census, with 24.6 percent living in low income [11]. These census estimates describe the broader service region and should not be interpreted as demographic characteristics of the clinic population itself. Given the rise in clinician-reported cases and the broader context of food insecurity in remote northern populations, this study addresses a central question: does vitamin C deficiency exist among those adults presenting for medical care in the La Ronge region, how is it patterned by clinical presentation and demographic profile, and how might broader social determinants of health help contextualize these findings? By integrating clinical and contextual data, the objective is to inform both clinical practice and policy aimed at reducing preventable micronutrient deficiencies in food-insecure populations.

## Methods

### Ethics statement

This study was conducted under the governance and approval of Lac La Ronge Indian Band Health Services (LLRIBHS) and the Northern Inter-Tribal Health Authority (NITHA), in accordance with OCAP (Ownership, Control, Access, and Possession) principles [12], which guided data ownership, community control of study processes, and decisions about data use and reporting. The University of Saskatchewan Research Ethics Board reviewed the project and exempted it as an external organizational report under TCPS2 Article 2.5 [13] (Certificates E-Bio-003 and E-Bio-077), and waived the requirement for individual informed consent. As this was a retrospective review of existing clinical records, data were accessed by JDCI in identifiable form for the purposes of data extraction and linkage, after which all data were fully de-identified prior to analysis. No other members of the research team had access to identifiable information. Although the study design allowed for inclusion of participants under 18 years of age, no children had serum ascorbic acid testing during the study period. Accordingly, parental or guardian consent was not required. Operational approval was granted by the Saskatchewan Health Authority (SHA) and partnering community organizations.

We conducted a retrospective descriptive analysis of electronic medical record (EMR) data from the La Ronge Medical Clinic (LRMC), which provides primary care services to residents of La Ronge, Air Ronge, and multiple remote First Nations communities in Northern Saskatchewan, Canada. The region faces longstanding structural inequities affecting food affordability, access, and nutritional quality, shaped by colonization, climate impacts, and geographic isolation. We included all individuals who had serum ascorbic acid (vitamin C) testing performed between January 1, 2011, and March 31, 2024. Records were eligible if they contained a documented serum ascorbic acid result, alongside demographic and clinical information. There were no exclusion criteria beyond missing test results. First Nations status was inferred from the presence of a treaty number in the EMR, recognizing that this proxy may misclassify some individuals.

We extracted patient demographics (age, sex, First Nations status), test indication, and serum ascorbic acid levels from structured and free‑text fields in the EMR. Test indications were manually reviewed from clinician notes. Two team members independently categorized indications, resolving discrepancies by consensus. Clinical indications were classified into dermatologic, oral, musculoskeletal, fatigue/weakness, or non-specific categories. For exploratory comparison, patients were categorized as having documented characteristic scurvy features if the testing indication included poor wound healing, characteristic hair or follicular abnormalities (e.g., corkscrew or flattened hairs or perifollicular hemorrhage), capillary fragility or petechial findings, or characteristic gingival abnormalities. Patients tested for other indications, including fatigue or weakness, musculoskeletal pain, poor dietary intake, anemia, or other non-specific concerns, were categorized as not having documented characteristic scurvy features. Smoking status was inconsistently documented and was therefore not analyzable.

Data extraction occurred between 13 May 2024 and 31 May 2024, as well as between 6 May 2025 and 15 May 2025. During data collection, the primary reviewer (JDCI) accessed identifiable information within the EMR to verify eligibility and link records. A master linkage file was used to manage identifiers during this stage. After extraction, all analyses were conducted using a de-identified dataset, with the linkage file stored separately and accessible only to the primary reviewer. No other authors had access to identifiable information during or after data collection.

Vitamin C deficiency was defined as ≤11 µmol/L, and hypovitaminosis C as 11–23 µmol/L, consistent with established laboratory thresholds. Normal vitamin C status was defined as ≥23 µmol/L.

Serum samples were collected by certified phlebotomists at the La Ronge Health Centre. Blood was collected in serum separator tubes, protected from light, chilled immediately, centrifuged to separate plasma, and transported on dry ice to the Roy Romanow Provincial Laboratory (RRPL) in Regina, Saskatchewan, over 600 kilometers away. Samples were stored at −70 °C until analysis, and processed according to the RRPL protocols.

We summarized demographic and clinical characteristics using descriptive statistics. Fisher’s exact test was used to compare deficiency prevalence by sex, age group, First Nations status, and presence or absence of documented characteristic scurvy features, due to small sample sizes. A p-value <0.05 was considered statistically significant. Analyses were limited to descriptive statistics appropriate for small samples. No multivariable modelling was undertaken given the sample size and the exploratory, descriptive purpose of the study.

The research team included clinicians, epidemiologists, and community health leaders with long-standing partnerships in Northern Saskatchewan. Indigenous team members from LLRIBHS guided study priorities, contributed to interpretation, and approved the final manuscript. Team members from NITHA collaborated in governance, methodological decisions, and manuscript writing. Non-Indigenous authors approached the work using a community-directed and decolonizing perspective grounded in local relationships.

All individual-level data are held by the SHA, with governance and access guided by LLRIBHS and NITHA in accordance with OCAP principles. Only aggregated and de-identified results were shared for publication. Aggregated data may be available upon reasonable request, subject to review and approval by LLRIBHS and NITHA.

## Results

Between January 2011 and March 2024, 51 serum ascorbic acid tests were documented at the LRMC, with 50 of them occurring between August 2023 and March 2024. During this latter time period, a total of 7,709 unique patients accessed care at the La Ronge Medical Clinic, of whom 50 (0.65%) underwent serum ascorbic acid testing. Testing was clinician-initiated based on clinical suspicion or perceived risk rather than systematic population screening. Documented indications included dermatologic or oral findings, musculoskeletal symptoms, fatigue or weakness, poor dietary intake, chronic or recalcitrant anemia, and other clinical concerns, as described below. Three results (5.9%) were unreadable due to testing error, including the sole test prior to 2023. Thus, all analyzable results were from August 2023 onward.

Among the 50 patients tested from August 2023 to March 2024, 30 (60%) were female and 20 (40%) were male, all aged 20 years or older. Thirty-seven patients (74%) were identified as First Nations based on having a treaty number listed on the EMR. Compared with the overall clinic population, patients who underwent vitamin C testing were more frequently female (60.0% vs 54.9%), and were disproportionately represented in the 30–49 year age groups (Table 1). Of the 48 analyzable results, 10 (20.8%) indicated hypovitaminosis C levels (11–23 µmol/L), and 27 (56.3%) met the laboratory threshold for vitamin C deficiency (<11 µmol/L).

**Table 1:** Comparison of clinic population and patients undergoing vitamin C testing, La Ronge Medical Clinic, August 2023–March 2024.

Characteristic	Clinic population (n = 7709)	Tested for vitamin C (n = 50)
Female, n (%)	4236 (54.9%)	30 (60%)
Male, n (%)	3473 (45.1%)	20 (40%)
20-29 years	1033 (13.4%)	4 (8%)
30-39 years	1217 (15.8%)	13 (26%)
40-49 years	980 (12.7%)	12 (24%)
50-59 years	983 (12.8%)	8 (16%)
60-69 years	881 (11.4%)	8 (16%)
70 years and over	722 (9.4%)	5 (10%)
First Nations, n (%)	Not available*	37 (74%)

* Aggregate clinic-level First Nations status was not available from aggregate EMR data.

Fig 1 illustrates the distribution of test outcomes by age group, with the highest frequency of testing and vitamin C deficiency observed in individuals aged 30–49 years. Fig 2 displays ascorbic acid test outcomes by sex and First Nations status. As shown in Fig 3, vitamin C deficiency was significantly more common among First Nations patients than non–First Nations patients (65.8% vs 20.0%; Fisher’s exact test, p = 0.014). In exploratory comparisons, no significant differences in vitamin C deficiency were detected by sex (55.6% among females vs 57.1% among males; Fisher’s exact test, p = 1.00) or age group (p = 0.86). Testing for other micronutrients was not routinely performed in this cohort.

**Fig 1:**
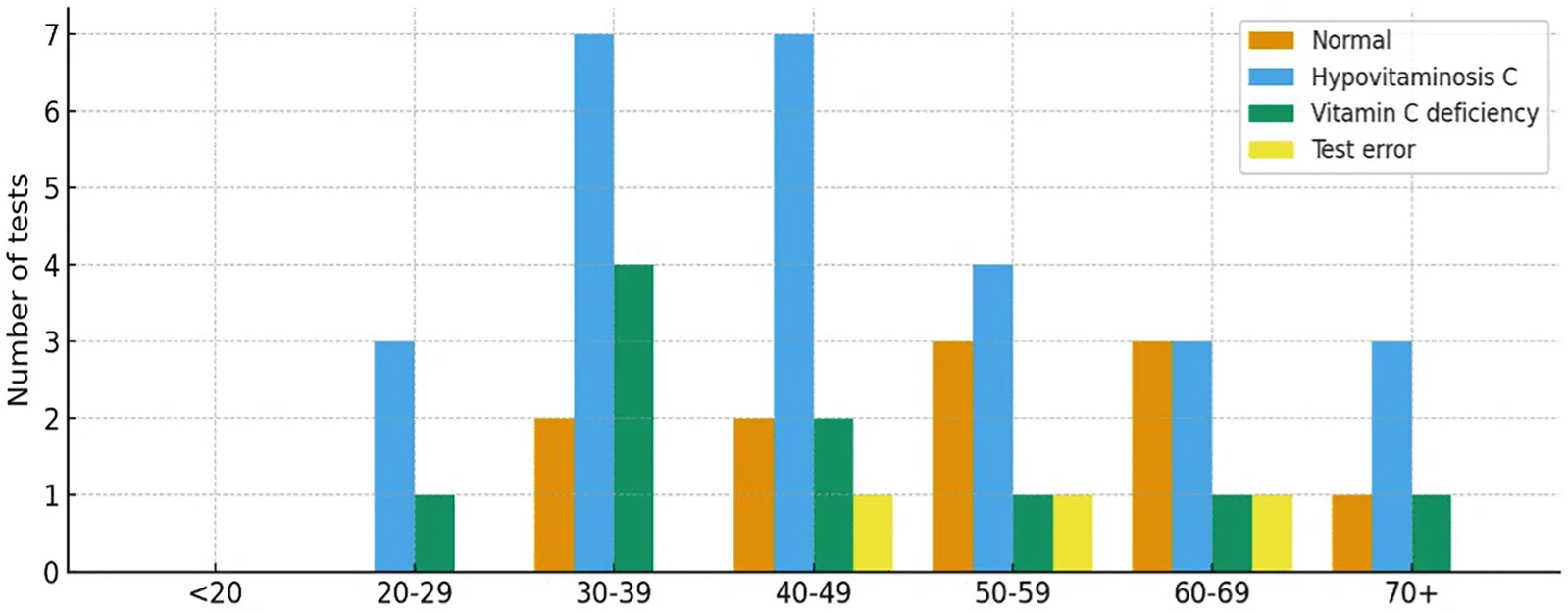
Distribution of ascorbic acid test results by age group. Bars show the number of patients tested in each age category, stratified by vitamin C status: deficient (<11 µmol/L), hypovitaminosis C (11–23 µmol/L), and normal (>23 µmol/L). Age categories are grouped in 10-year intervals.

**Fig 2:**
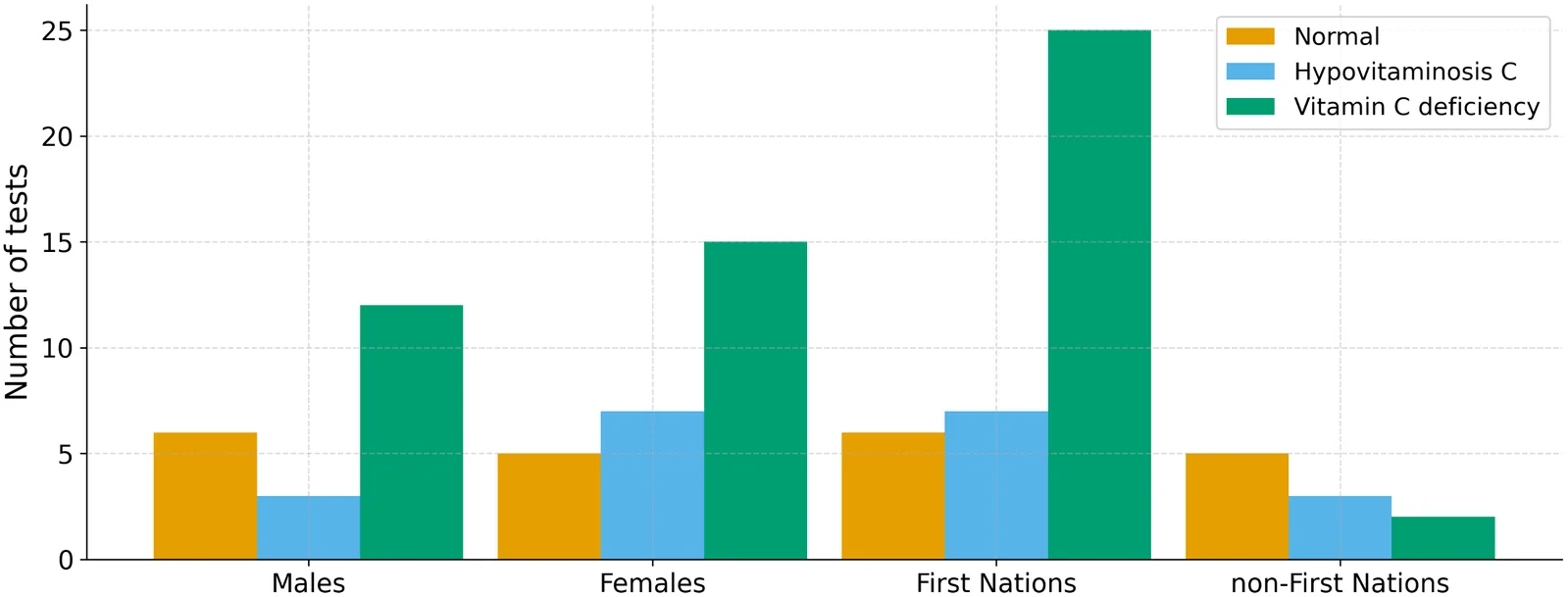
Ascorbic acid test outcomes by sex and First Nations status. Stacked bars illustrate the proportion of patients in each demographic group with vitamin C deficiency (<11 µmol/L), hypovitaminosis C (11–23 µmol/L), or normal levels (>23 µmol/L). Groups include males, females, First Nations, and non–First Nations patients.

**Fig 3:**
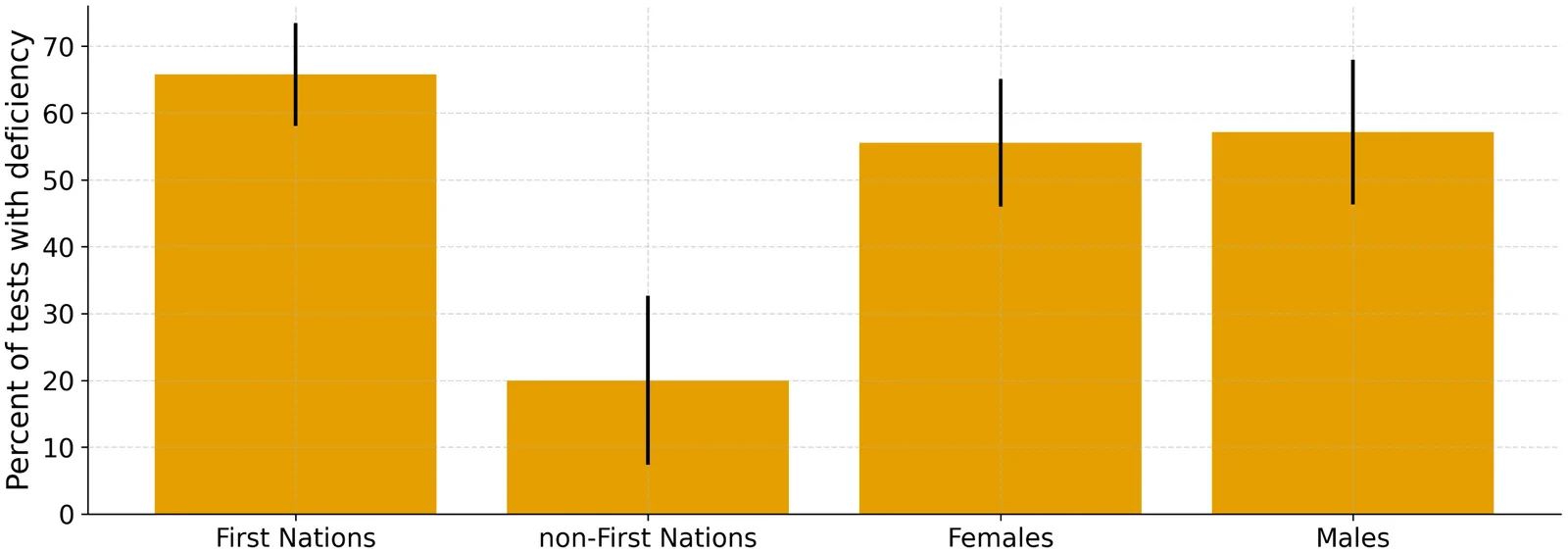
Vitamin C deficiency (<11 µmol/L) rates by sex and First Nations status. Bars display the percentage of patients within each demographic group who met laboratory criteria for vitamin C deficiency. Statistical comparison between First Nations and non–First Nations groups was performed using Fisher’s exact test.

Test indications were extracted from clinician notes and reflect the main documented reason for ordering serum ascorbic acid testing rather than a comprehensive inventory of all presenting symptoms. The most common documented clinical indications prompting clinician-initiated testing included dermatologic signs such as bruising or poor wound healing (21.6%, n = 11), oral health issues including gingival swelling or poor dentition (19.6%, n = 10), and musculoskeletal pain (11.8%, n = 6), followed by fatigue or weakness (9.8%, n = 5). Indications that were not specific for ascorbic acid deficiency comprised the remainder, including a history of poor food intake in tobacco smokers, and presentations in patients with chronic or recalcitrant anemia or diabetes. Among the 48 patients with analyzable vitamin C results, 11 had documented characteristic scurvy features and 37 did not. Vitamin C deficiency was identified in 7 of 11 patients (63.6%) with characteristic features and 20 of 37 patients (54.1%) without characteristic features. This difference was not statistically significant (Fisher’s exact test, p = 0.73).

## Discussion

Although vitamin C deficiency is often considered rare in high-income countries, it persists among clinically and socially vulnerable populations. Case reports, hospital-based series, and recent reviews from Australia, Europe, and North America describe deficiency among individuals with restricted diets, chronic illness, substance use, mental health conditions, and social isolation [2,3,14]. Population-level data further demonstrate that subclinical vitamin C deficiency persists in the general population. For example, Canadian Health Measures Survey data found measurable rates of deficiency and hypovitaminosis C among adults, particularly among those with lower socioeconomic status and smoking exposure [15].

Within this broader context, our findings suggest that vitamin C deficiency is an important clinical concern in Northern Saskatchewan, particularly among the First Nations patients tested. This pattern may reflect broader nutritional inequities related to poverty, food insecurity, and limited access to healthy foods [3,16]. The high proportion of deficient results raises concern that micronutrient deficiencies may be under-recognized in this population. Improving nutrition therefore requires addressing foundational determinants of health like food security, housing, and income stability. Indigenous worldviews emphasize relational wellness and cultural continuity, and interventions must therefore respect Indigenous knowledge systems and community-defined priorities [17].

These risks may be further exacerbated by reliance on ultra-processed foods, which are energy-dense but micronutrient-poor [10]. Evidence from Indigenous communities internationally, paralleling findings among First Nations in Canada, demonstrates that micronutrient deficiencies are closely linked to historical displacement, erosion of land-based food systems, and inequitable food pricing structures [17–21]. In northern Saskatchewan, these forces likely contribute through high food costs, restricted local food availability, climate-driven changes to harvesting, and an increasing reliance on market foods that are calorie-dense but nutrient-poor [18,20–22]. These patterns can be further understood within a conceptual framework linking structural determinants to micronutrient deficiency.

The pathways linking structural inequities to vitamin C deficiency can be understood through a social determinants of health framework, in which upstream factors including poverty, food insecurity, geographic isolation, and the ongoing impacts of colonization shape access to nutritious foods and dietary patterns. Within this framework, vitamin C deficiency may be one biological consequence of limited access to fresh or minimally processed foods. Our study cannot establish this causal pathway, but the findings are consistent with structural and environmental conditions influencing diet and, in turn, micronutrient status.

Colonial disruption of Indigenous food systems continues to shape the nutritional landscape in northern Canada [18,22]. Traditional harvesting, preservation, and land-based food practices have been eroded by historic policies restricting land access, climate change impacts, and market-driven food systems [18,22]. These structural forces have produced persistent food insecurity, particularly in remote First Nations communities [17,21,22]. Data from the First Nations Food, Nutrition and Environment Study (FNFNES) (2008–2018) show that vitamin C intake among First Nations adults across Canada was frequently below recommended levels, particularly among smokers and older adults [22]. In the Lac La Ronge Indian Band cohort, half reported no vitamin C–containing foods in a 24-hour recall. High rates of tobacco use in northern Saskatchewan [22] further increase vitamin C requirements [3], compounding dietary vulnerability. These findings reinforce the nutritional risks observed in our clinical data and highlight persistent barriers to accessing micronutrient-rich foods, even in communities with strong traditional harvesting knowledge. Addressing these inequities will require restoring access to culturally important plant foods and supporting community-led food initiatives that strengthen local food sovereignty, including revitalizing home and community gardening and traditional preservation practices where desired.

The high proportion of vitamin C deficiency among patients tested in this study mirrors broader patterns of food insecurity and micronutrient deficiency documented among Indigenous communities across Canada [17,22]. Food insecurity remains a public health crisis, contributing to long-term physical, cognitive, and emotional health challenges [17]. These realities emphasize the importance of Indigenous-led, culturally anchored food strategies that restore access to traditional foods, empower community leadership, and address poverty as a root cause of malnutrition [18,19]. Over 60% of FNFNES respondents identified climate change, land access restrictions, and loss of traditional knowledge as key impediments to harvesting activities [22]. These findings underscore the role of disrupted food systems in driving micronutrient deficiencies and the need for interventions that restore both food access and intergenerational food knowledge.

Existing federal programs such as *Nutrition North Canada* aim to lower the cost of perishable foods in isolated communities [23]. While the program has modestly increased the availability of some items like milk and fresh produce, evaluations show mixed results in affordability and transparency, with subsidies not always passed on to consumers, particularly where retail competition is limited [20,21]. Moreover, the program does little to support culturally relevant foods or strengthen local food sovereignty [21]. Complementary community-led strategies are therefore needed to ensure that fresh, nutrient-rich foods are affordable, culturally appropriate, and treated not merely as commodities but as basic rights.

Accurate assessment of serum ascorbic acid is technically challenging, particularly in remote regions. Samples require protection from light, rapid processing, and frozen transport to minimize degradation, creating potential for falsely low results if handling is suboptimal [24]. Interpretation is further complicated by inflammation, which can transiently lower circulating vitamin C concentrations independent of tissue stores [25]. Although local laboratory protocols are rigorous, these pre-analytical and biological factors introduce some uncertainty into serum ascorbic acid measurements. However, considered alongside the clinical presentations, dietary context, and parallel findings in Indigenous populations, the high proportion of deficient results in this clinically selected sample is unlikely to be explained by laboratory artifact alone.

Due to cost and technical complexity, widespread screening in high-risk areas may not be feasible. Ascorbic acid testing costs $15.78 CAD per sample excluding specialized transport requirements [26]. In contrast, empiric treatment with a full course of oral vitamin C (e.g., 500 mg daily for one month) costs less than $2.50 CAD per patient [27] and is safe, well-tolerated, and rapidly effective [28]. For many rural and remote clinics, particularly those without on-site laboratory capacity, empiric supplementation may be more practical than confirmatory testing for patients with suggestive symptoms, poor dietary intake, smoking history, or socioeconomic risk factors. This approach is supported by the low cost and safety of oral vitamin C and is consistent with recommendations to initiate treatment when deficiency is suspected but testing is unavailable [29].

Serum ascorbic acid testing increased sharply after August 2023, likely reflecting increased local clinical suspicion and targeted testing. The high proportion of deficient results among those tested nevertheless suggests that vitamin C deficiency is a clinically relevant problem in this setting. Most deficient individuals were adults in their 30s and 40s, though results were based on clinical indication rather than population testing. This pattern may reflect testing practices and is broadly consistent with national data showing higher risk among adults aged 40–59 years [15]. In our context, co-occurring factors such as smoking, diet quality, and socioeconomic disadvantage may contribute. Given the small sample size, these exploratory comparisons should not be interpreted as evidence of age- or sex-specific differences in deficiency. Larger population-based studies, including youth, are needed to evaluate whether such patterns exist.

Characteristic scurvy features, including impaired wound healing, perifollicular hemorrhage, corkscrew hairs, capillary fragility, and gingival abnormalities, remain important clinical triggers for considering vitamin C deficiency [30]. However, deficiency was also common among patients tested without these characteristic features. Non-specific presentations, including fatigue, weakness, or musculoskeletal pain, may therefore warrant consideration of vitamin C deficiency when accompanied by dietary or social risk factors. Given the small, clinically selected sample, our findings cannot establish the diagnostic sensitivity of these symptoms or support their use as independent screening criteria. Further research is needed to determine which combinations of symptoms and risk factors best identify patients who may benefit from testing or empiric supplementation.

Addressing vitamin C deficiency in northern Saskatchewan requires more than clinical recognition. Sustainable solutions must be Indigenous-led, rooted in food sovereignty, and focused on rebuilding local food systems. Community priorities include revitalizing land-based harvesting, supporting home and community gardens, improving traditional food preservation practices, and strengthening school- and community-based nutrition programs. LLRIBHS and NITHA, for example, have already integrated these findings into clinical guidance and nutrition programming. Broader upstream actions, such as eliminating poverty, improving housing, supporting climate resilience, and reforming Nutrition North Canada, remain essential to long-term nutritional well-being.

While the findings have important clinical and public health implications, the present study was not designed to evaluate the effectiveness or cost-effectiveness of specific interventions. Future research should aim to establish population-level prevalence of vitamin C deficiency and identify high-risk subgroups within northern and Indigenous communities. Such data would enable more precise targeting of interventions and support formal cost-benefit analyses of potential strategies, including screening, supplementation, and improvements to food access. In this context, intervention development should be guided by community priorities and Indigenous knowledge systems, ensuring that approaches are both evidence-informed and culturally appropriate.

### Limitations

This study has several limitations that must be considered when interpreting its findings. First, despite adherence to recommended laboratory protocols, serum ascorbic acid is highly labile and susceptible to oxidation [24]. Transport from remote locations introduces risk for pre-analytical degradation, potentially resulting in falsely low levels. Second, samples were collected in non-fasting conditions, which may lead to modestly higher serum levels and therefore underestimation of deficiency prevalence [25,28]. Third, markers of inflammation, such as C-reactive protein, were not consistently ordered alongside vitamin C testing. Acute or chronic inflammation can transiently reduce circulating vitamin C, complicating interpretation [25]. Fourth, First Nations status was inferred through treaty number documentation in the EMR, which may have resulted in misclassification and underrepresentation of some Indigenous individuals. Aggregate clinic-level First Nations status was unavailable, preventing assessment of whether the tested cohort was representative of the clinic population with respect to First Nations status. Fifth, smoking status and diet quality, which are important determinants of vitamin C requirements [3], were inconsistently documented and could not be analyzed. Sixth, the study relied on clinician-initiated testing, resulting in a convenience sample of individuals selected based on clinical suspicion or perceived risk. This introduces selection bias and limits the representativeness of the sample relative to the broader clinic population or community. As a result, the proportion of vitamin C deficiency observed in this study should not be interpreted as a population-level prevalence estimate. Additionally, clinical features were derived from the documented indication for testing rather than systematic symptom assessment, which may have resulted in misclassification of patients with or without characteristic scurvy features.

Finally, the retrospective design and small sample size preclude causal inference and limit the precision of subgroup comparisons. The findings, particularly comparisons by age and sex and interpretations regarding underlying drivers, should therefore be considered exploratory and hypothesis-generating rather than definitive. Despite these limitations, the study offers important insight into a largely undocumented micronutrient issue in northern Saskatchewan and highlights the structural determinants shaping dietary inequities.

### Recommendations

These findings suggest several potential directions for policy and practice, although further research is needed to inform their effectiveness and cost-effectiveness.

1. Strengthen Indigenous Food Sovereignty: Strengthening Indigenous food sovereignty may support improved micronutrient intake and overall health. Potential approaches include supporting Indigenous-led initiatives that revitalize traditional harvesting, cultivation, preservation, and intergenerational food knowledge. This could include expanding home and community gardens, microgreen production, and culturally grounded food education in schools. Sustained funding for community-controlled food programs will likely be important, although further evaluation is needed to assess their impact on nutritional outcomes.
2. Reform Nutrition North Canada: Policy-level interventions such as reforming Nutrition North Canada may help address structural barriers to accessing nutritious foods. Potential areas for improvement include enhancing transparency and accountability to ensure subsidies benefit consumers directly, and strengthening support for culturally relevant foods, including country foods and land-based harvesting. Further research is needed to evaluate how such policy changes influence dietary patterns and micronutrient status.
3. Expand Clinical and Public Health Responses: Clinical and public health responses could be expanded to improve identification and management of vitamin C deficiency in high-risk populations. Potential strategies include incorporating dietary screening into primary care, considering empiric vitamin C supplementation for high-risk individuals, and developing training to support recognition of micronutrient deficiency. Community-based programs, such as targeted multivitamin distribution, may also be beneficial, though their effectiveness and cost-effectiveness require further evaluation.
4. Address Structural Determinants: Addressing structural determinants of health may be necessary to achieve sustained improvements in nutrition. This could include policies aimed at reducing poverty, improving housing stability, and increasing access to affordable food. Enhancements to transportation infrastructure, storage capacity, and northern supply chains may help reduce cost barriers associated with fresh foods. The impact of such interventions on micronutrient status warrants further study.
5. Advance Community-Based Research and Monitoring: Further research is needed to better define the burden and distribution of vitamin C deficiency and to inform targeted interventions. This includes establishing population-level prevalence, identifying high-risk subgroups, and evaluating the effectiveness and cost-effectiveness of potential interventions. Expanding Indigenous-led nutrition surveillance systems and participatory research methods that reflect OCAP principles will be important to ensure future work is both scientifically robust and community-informed.
