# Supplementary material for: Vitamin C deficiency in a Northern Saskatchewan Population, Canada
Source: PLOS Glob Public Health. 2026 Oct 28;6(10):e0007464. doi: 10.1371/journal.pgph.0007464 (PMC13716197; doi:10.1371/journal.pgph.0007464)
Supplement: Supplementary file 1 [file pgph.0007464.s003.pdf]

Dear Editors,

We thank you and Reviewer 1 for the thoughtful and constructive feedback on our manuscript, “Vitamin C Deficiency in a Northern Saskatchewan Population, Canada.” We have revised the manuscript accordingly and believe these changes have substantially strengthened the scientific rigor, clarity, and policy relevance of the paper.

Below we provide a point-by-point response to the editorial and reviewer comments.

Editorial Comments

1. Please provide additional details regarding participant consent, including whether data were anonymized before access and whether consent was waived.

We have revised the Ethics Statement to explicitly state that the University of Saskatchewan Research Ethics Board reviewed the project and exempted it as an external organizational report under TCPS2 Article 2.5, and waived the requirement for individual informed consent. We now clarify that identifiable clinical records were accessed only by the primary reviewer (JDCI) for the purposes of eligibility verification and linkage during data extraction, after which all data were fully de-identified prior to analysis. No other members of the research team had access to identifiable information.

2. Please provide a completed Competing Interests statement.

We have revised the Competing Interests statement to use the journal’s required wording: “The authors have declared that no competing interests exist.”

3. Please remove references to unpublished data or include these data in supplementary material.

We have removed the previously cited unpublished provincial laboratory comparison data from the manuscript in full, in keeping with PLOS data access policy. This paragraph has been replaced with a more conservative interpretation based solely on the presented La Ronge Medical Clinic dataset. No unpublished external data are referenced in the revised manuscript.

4. Please remove trademark/copyright symbols, including OCAP®.

We removed trademark symbols from the manuscript text, including all uses of “OCAP®,” which now appears as “OCAP” throughout the revised clean manuscript in accordance with journal style.

5. Please revise the Data Availability Statement to comply with PLOS requirements for third-party data.

We revised the Data Availability Statement to fully align with PLOS requirements for third-party data. The revised statement now:

- describes the dataset and its source (de-identified clinical EMR data from the La Ronge Medical Clinic held by the Saskatchewan Health Authority),
- confirms that formal permission to access and use the data was obtained through Saskatchewan Health Authority operational approval and governance approvals from Lac La Ronge Indian Band Health Services (LLRIBHS) and the Northern Inter-Tribal Health Authority (NITHA),
- states explicitly that the authors received no special privileges in accessing these data, and
- provides the information required for other researchers to apply for access through the Saskatchewan Health Authority, with note that additional review may be required through LLRIBHS and NITHA in accordance with OCAP and Indigenous data governance principles.

Reviewer #1

We thank Reviewer 1 for their thoughtful and constructive review. We have revised the manuscript in an attempt to address each of the concerns raised.

1. The manuscript would benefit from reference to recent literature describing vitamin C deficiency in clinical populations in high-income countries and non-Indigenous populations.

We agree and have revised the Discussion accordingly. We now explicitly situate our findings within contemporary high-income clinical literature by adding recent hospital-based and scoping review evidence from Australia, Europe, and North America, including the recent scoping review by Golder et al. (Nutr Rev 2024), which addresses vitamin C deficiency in adult hospitalized patients in high-income settings. We also strengthened comparison to non-Indigenous population data by referencing Canadian Health Measures Survey findings on vitamin C deficiency and hypovitaminosis C among Canadian adults. These additions provide a more contemporary and internationally relevant epidemiologic context for our findings.

2. The sample is a small convenience sample and cannot provide a population prevalence estimate.

We agree and have revised the manuscript throughout to better reflect this limitation. We now explicitly describe the cohort as a clinician-initiated convenience sample selected on clinical suspicion, emphasize that the observed proportion of deficiency should not be interpreted as a population-level prevalence estimate, and repeatedly frame the findings as reflecting burden among tested patients rather than prevalence in the clinic or community population.

3. It would strengthen the study to compare the tested sample with the clinic population as a whole, including what proportion of clinic patients were tested.

We agree and have added this comparison. We now report that 6,342 unique patients accessed care at the La Ronge Medical Clinic during the August 2023–March 2024 study interval, of whom 50 (0.79%) underwent serum ascorbic acid testing. We also added Table 1 comparing the clinic population and tested cohort by sex, and we now note that those tested were somewhat more likely to be female than the clinic population overall. Aggregate clinic-level Indigenous identity data were not available and this is now stated explicitly. We hope these additions provide better context for understanding representativeness and selection bias.

4. It would be reasonable to exclude the sole pre-2023 test and focus on the contemporary testing period.

We appreciate this suggestion. Rather than excluding the test entirely, we retained it in the full sampling frame for transparency, but clarified that the sole pre-2023 test was unreadable and therefore not analyzable. We now explicitly state that all analyzable results were from August 2023 onward. We hope this preserves transparency regarding the full study period while ensuring that interpretation is focused on the contemporary clinical period.

5. Claims regarding causation were stronger than the data support.

We agree and substantially revised the Discussion to better distinguish observation from inference. Causal statements have been softened throughout, and we now frame proposed pathways linking structural inequities to vitamin C deficiency as theory-informed and hypothesis-generating rather than causal conclusions established by the data. We also added explicit language stating that the study was not designed to evaluate causal relationships and that interpretations regarding structural drivers should be considered hypothesis-generating.

6. Recommendations were reasonable but should be framed more cautiously given the study design.

We agree and revised the Recommendations section accordingly. Recommendations are now framed more explicitly as potential directions for policy and practice rather than evidence-tested interventions. We also added clearer language emphasizing that further research is needed to determine effectiveness and cost-effectiveness of proposed strategies, including screening, supplementation, and food-system interventions.

7. It would be beneficial to include more detail on patients with non-classical manifestations.

We appreciate this suggestion and addressed it by expanding the Results section to include a descriptive comparison of patients with classical versus non-classical clinical presentations prompting vitamin C testing. We now report that 13 patients lacked classical signs or symptoms, that 31% of these patients were vitamin C deficient, that they were older on average, and that

they had higher mean vitamin C levels and lower deficiency yield than those with classical presentations. Given the small subgroup sizes and descriptive nature of these comparisons, we elected to present these findings narratively rather than in an additional table to avoid overinterpretation of unstable subgroup estimates.

We thank the editor and reviewer again for their thoughtful and constructive feedback. We believe the revised manuscript is substantially stronger because of these revisions and hope it is now suitable for publication in PLOS Global Public Health.

Sincerely,

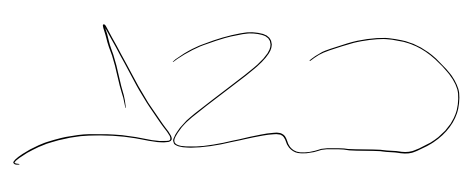

Jeffrey D.C. Irvine, corresponding author, on behalf of all authors
