# Supplementary material for: Vitamin C deficiency in a Northern Saskatchewan Population, Canada
Source: PLOS Glob Public Health. 2026 Oct 28;6(10):e0007464. doi: 10.1371/journal.pgph.0007464 (PMC13716197; doi:10.1371/journal.pgph.0007464)
Supplement: Supplementary file 2 [file pgph.0007464.s004.pdf]

Response to Reviewer #2

We thank the reviewer for their thoughtful comments and suggestions. We have revised the manuscript accordingly and believe these changes have improved the clarity and rigor of the paper. Our responses are provided below.

Comment 1:
"If Indigenous Identity is not available why offer the percentage of 83.9%? Where did this percentage come from? Is it truly reasonable to assume that the clinic population directly mirrors the population in that area?"

Response:
Thank you for this important observation. We agree that the original wording could have implied that regional census demographics reflected the clinic population. We have revised the manuscript to clarify that the 83.9% figure is derived from the 2021 Statistics Canada Census and refers to the broader service region rather than the clinic population itself. We now explicitly state in the Introduction that these census estimates should not be interpreted as demographic characteristics of the clinic population. In the Results section, we removed the comparison between the tested cohort and the regional Indigenous population and instead report only the observed proportion of First Nations patients within the tested cohort. We also revised Table 1 and the Limitations section to clarify that aggregate clinic-level First Nations status was unavailable, preventing assessment of whether the tested cohort was representative of the clinic population with respect to First Nations status.

Comment 2:
"The part about smoking status should be in the methods rather than the results if you were unable to assess."

Response:
We agree and have moved this information to the Methods section. The manuscript now states that smoking status was inconsistently documented in the electronic medical record and was therefore not analyzable. The corresponding statement has been removed from the Results section.

Comment 3:
"The wording did not reach statistical significance implies to some readers that it may have been close to significance when it was not. I would suggest the wording 'this difference was not statistically significant.'"

Response:
Thank you for this suggestion. We have revised the wording accordingly. The Results section now states that the observed difference "was not statistically significant" and provides the associated Fisher's exact test p-value.

Comment 4:
"Methods/Results: Where are the p-results for each paired t-test? I see you report some (e.g.,

Line 177) but it's unclear what the p-values are for each paired t-test. I'd recommend reporting these, as it's hard for the reader to interpret what differences were tested and what had significance based on the way it is currently written."

Response:
We appreciate this comment and have clarified the statistical analyses throughout the manuscript. The study did not employ paired t-tests; rather, Fisher's exact tests were used because of the small sample size and categorical nature of the data. We have revised the Methods section to explicitly state all comparisons evaluated using Fisher's exact test, including comparisons by First Nations status, sex, age group, and presence or absence of classical symptoms. We have also reported the corresponding p-values in the Results section, including for comparisons by First Nations status, sex, age group, and classical versus non-classical symptom presentation.

We thank the reviewer again for these constructive comments, which we believe have improved the clarity and transparency of the manuscript.

Corresponding author, on behalf of all authors
