# Supplementary material for: Vitamin C deficiency in a Northern Saskatchewan Population, Canada
Source: PLOS Glob Public Health. 2026 Oct 28;6(10):e0007464. doi: 10.1371/journal.pgph.0007464 (PMC13716197; doi:10.1371/journal.pgph.0007464)
Supplement: Supplementary file 3 [file pgph.0007464.s002.docx]

**PGPH-D-25-03694R1**

Vitamin C Deficiency in a Northern Saskatchewan Population, Canada

- Line 182: If Indigenous Identity is not available why offer the percentage of 83.9%? Where did this percentage come from? Is it truly reasonable to assume that the clinic population directly mirrors the population in that area?
- Line 209: The part about smoking status should be in the methods rather than the results if you were unable to assess.
- Line 214: the wording did not reach statistical significance implies to some readers that it may have been close to significance when it was not. I would suggest the wording “this difference was not statistically significant.”
- Methods/Results: Where are the p-results for each paired t-test? I see you report some (e.g., Line 177) but it’s unclear what the p-values are for each paired t-test. I’d recommend reporting these, as it’s hard for the reader to interpret what differences were tested and what had significance based on the way it is currently written.
