# Supplementary material for: Vitamin C deficiency in a Northern Saskatchewan Population, Canada
Source: PLOS Glob Public Health. 2026 Oct 28;6(10):e0007464. doi: 10.1371/journal.pgph.0007464 (PMC13716197; doi:10.1371/journal.pgph.0007464)
Supplement: Supplementary file 4 [file pgph.0007464.s005.docx]

Dear Editor and Reviewers,

Thank you for the thoughtful review of our revised manuscript, “Vitamin C Deficiency in a Northern Saskatchewan Population, Canada.” We appreciate the reviewers’ constructive comments, which we believe helped us further clarify the study design, appropriately frame the limitations of this clinically selected sample, and improve the readability and clinical interpretation of the manuscript.

We have addressed each comment as outlined below.

**Reviewer #1**

**Comment:**
The revised manuscript addresses all issues noted in the peer review. The only minor suggestion is regarding Line 73: “anecdotal reports from physicians...” where it might be helpful to specify which physicians reported cases of scurvy, i.e. “anecdotal reports from local physicians...”.

**Response:**
Thank you for this suggestion. We have clarified that these anecdotal reports arose from local clinicians. The Introduction now states, “In recent years, anecdotal reports from local clinicians raised suspicions of scurvy.” The following sentence provides further context by identifying the family physicians at the La Ronge Medical Clinic and nurses at surrounding outpost stations who provide care within the region.

**Reviewer #3**

We thank the reviewer for recognizing the study’s focus on health equity, adherence to OCAP principles, and relevance to policy and practice. We appreciate the additional comments and have made several changes to better distinguish findings from this clinically selected sample from population-level estimates, clarify the exploratory nature of the analyses, and strengthen the interpretation of clinical presentations.

**Comment 1**

**The use of a convenience sample based on clinical suspicion introduces significant selection bias. The 53% deficiency rate likely reflects a high-risk subgroup rather than the general population. This limitation must be explicitly stated in the Abstract and Results to avoid misleading readers.**

**Response:**
We agree and have revised the manuscript to make the clinically selected nature of the sample explicit.

In the Abstract Methods, we now state that serum ascorbic acid testing was “clinician-initiated” and “based on clinical suspicion or perceived risk rather than population-based screening.” The Abstract Interpretation now explicitly states: “These findings reflect a clinically selected subgroup and should not be interpreted as population-level prevalence.”

We have similarly clarified this in the Results. We report that, among 7,709 unique patients accessing the clinic during the period of concentrated testing, only 50 (0.65%) underwent serum ascorbic acid testing, and that testing was clinician-initiated based on clinical suspicion or perceived risk rather than systematic population screening.

The Limitations section has also been strengthened to state that clinician-initiated testing resulted in a convenience sample, introduces selection bias, and limits representativeness relative to the broader clinic population and community. We explicitly state that the proportion with vitamin C deficiency in this study should not be interpreted as a population-level prevalence estimate.

**Comment 2**

**Due to the small sample size (n=48), the authors should avoid over-interpreting P-values for age and sex. The study should be framed as “exploratory” or “hypothesis-generating” rather than definitive.**

**Response:**
We agree. We have revised the manuscript throughout to emphasize the exploratory nature of these analyses.

The Abstract Methods now describes the study as an “exploratory retrospective EMR-based review.” In the Methods, we clarify that analyses were limited to descriptive statistics appropriate for the small sample and that no multivariable modelling was undertaken given the sample size and exploratory, descriptive purpose of the study.

In the Results, age- and sex-based analyses are explicitly described as “exploratory comparisons.” In the Discussion, we state that these comparisons should not be interpreted as evidence of age- or sex-specific differences in vitamin C deficiency. Finally, the Limitations section now states that the small sample limits the precision of subgroup comparisons and that findings, particularly those concerning age, sex, and potential underlying drivers, should be considered exploratory and hypothesis-generating rather than definitive.

**Comment 3**

**While the 31% deficiency rate among asymptomatic patients is clinically significant, the authors should discuss whether “non-classical” symptoms (e.g., fatigue or joint pain) could serve as more sensitive screening triggers for this population.**

**Response:**
Thank you for highlighting this clinically important issue. In response to this comment, we revisited and refined our exploratory analysis of clinical presentations. We clarified the definition of characteristic scurvy features in the Methods and restricted this comparison to the 48 patients with analyzable vitamin C results.

Patients were classified as having documented characteristic scurvy features when the testing indication included findings such as poor wound healing, characteristic hair or follicular abnormalities, capillary fragility or petechial findings, or characteristic gingival abnormalities. Among the 48 patients with analyzable results, 11 had documented characteristic scurvy features and 37 did not. Vitamin C deficiency was identified in 7 of 11 (63.6%) patients with characteristic features and 20 of 37 (54.1%) without characteristic features. This difference was not statistically significant (Fisher’s exact test, p=0.73). The manuscript has been updated to report this refined analysis in place of the previously reported 31% estimate.

We have also expanded the Discussion to address the reviewer’s underlying clinical question. We now note that vitamin C deficiency was common among patients tested without characteristic scurvy features and that non-specific presentations such as fatigue, weakness, or musculoskeletal pain may warrant consideration of vitamin C deficiency when accompanied by dietary or social risk factors. However, given the small and clinically selected sample, we emphasize that our findings cannot establish the diagnostic sensitivity of these symptoms or support their use as independent screening criteria. We suggest that future research examine which combinations of symptoms and dietary or social risk factors best identify patients who may benefit from testing or empiric supplementation.

**Comment 4**

**Given the stark contrast between the clinic population (n=7,709) and the tested group (n=50), the authors should explain the clinical criteria or specific triggers used to select this small fraction (0.64%) of patients in the Results section.**

**Response:**
We agree that this required greater clarification. We have revised the Results to state explicitly that testing was clinician-initiated based on clinical suspicion or perceived risk rather than systematic population screening. We also describe the documented indications for testing, which included dermatologic and oral findings, musculoskeletal symptoms, fatigue or weakness, poor dietary intake, chronic or recalcitrant anemia, and other clinical concerns.

We further clarify that the test indication represents the main documented reason for ordering serum ascorbic acid testing rather than a comprehensive inventory of presenting symptoms. The most frequently documented indications were dermatologic findings such as bruising or poor wound healing, oral health concerns such as gingival swelling or poor dentition, musculoskeletal pain, and fatigue or weakness.

**Comment 5**

**While the explanation of serum ascorbic acid testing challenges is excellent, the section is overly long. I suggest streamlining this part or moving the technical details to “Supplementary Material” to improve the Discussion's flow.**

**Response:**
Thank you. We agree that the detailed discussion of serum ascorbic acid testing interrupted the flow of the Discussion. We have substantially streamlined this section while retaining the key considerations regarding pre-analytical degradation and the effects of inflammation on circulating vitamin C concentrations.

Detailed sample handling procedures remain described in the Methods, including protection from light, immediate chilling, centrifugation, frozen transport, and storage prior to analysis. The Discussion now focuses only on those aspects of serum testing that are necessary for interpreting the study findings. We therefore did not add supplementary material, as the essential methodological information is already provided in the manuscript.

**Comment 6**

**Although the English is high quality, some paragraphs in the Discussion are overly complex. A final polish for readability is recommended to better engage a broader global public health audience.**

**Response:**
Thank you. We have completed a final readability edit of the Discussion. Several complex sentences have been simplified, repetitive passages have been removed or consolidated, and the discussion of structural determinants, empiric supplementation, clinical testing, and increased local testing has been streamlined.

We have aimed to improve accessibility for a broader global public health readership while preserving the manuscript’s emphasis on Indigenous health equity, food sovereignty, structural determinants of nutrition, and community-defined priorities.

We thank the reviewers again for their careful and constructive feedback. We believe these revisions have substantially improved the clarity, methodological transparency, and interpretation of the manuscript.

Sincerely,

Jeffrey D.C. Irvine
Corresponding author, on behalf of the authors
