# Supplementary material for: Vitamin C deficiency in a Northern Saskatchewan Population, Canada
Source: PLOS Glob Public Health. 2026 Oct 28;6(10):e0007464. doi: 10.1371/journal.pgph.0007464 (PMC13716197; doi:10.1371/journal.pgph.0007464)
Supplement: Peer review history

# Peer review: Decision Letter 0

**DOI:** 10.1371/journal.pgph.0007464.r001

**Authors:** Ilse Bloom

16 Mar 2026

PGPH-D-25-03694

Vitamin C Deficiency in a Northern Saskatchewan Population, Canada

PLOS Global Public Health

Dear Dr. Irvine,

Thank you for submitting your manuscript to PLOS Global Public Health. After careful consideration, we feel that it has merit but does not fully meet PLOS Global Public Health’s publication criteria as it currently stands. Therefore, we invite you to submit a revised version of the manuscript that addresses the points raised during the review process.

Please note that we have only been able to secure a single reviewer to assess your manuscript. We are issuing a decision on your manuscript at this point to prevent further delays in the evaluation of your manuscript. Please be aware that the editor who handles your revised manuscript might find it necessary to invite additional reviewers to assess this work once the revised manuscript is submitted. However, we will aim to proceed on the basis of this single review if possible.

The comments from the reviewer can be found below. Please review these comments and make the appropriate revisions to your manuscript alongside a point-by-point response to the concerns raised.

Please submit your revised manuscript by Apr 30 2026 11:59PM. If you will need more time than this to complete your revisions, please reply to this message or contact the journal office at globalpubhealth@plos.org. When you're ready to submit your revision, log on to https://www.editorialmanager.com/pgph/ and select the 'Submissions Needing Revision' folder to locate your manuscript file.

Please include the following items when submitting your revised manuscript:

- A letter that responds to each point raised by the editor and reviewer(s). You should upload this letter as a separate file labeled 'Response to Reviewers'.
- A marked-up copy of your manuscript that highlights changes made to the original version. You should upload this as a separate file labeled 'Revised Manuscript with Track Changes'.
- An unmarked version of your revised paper without tracked changes. You should upload this as a separate file labeled 'Manuscript'.

Guidelines for resubmitting your figure files are available below the reviewer comments at the end of this letter.

We look forward to receiving your revised manuscript.

Kind regards,

Ilse Bloom

Staff Editor

PLOS Global Public Health

**Journal Requirements:**

1. Please provide additional details regarding participant consent. As you are reporting a retrospective study of medical records or archived samples, please ensure that you have discussed whether all data were fully anonymized before you accessed them and/or whether the IRB or ethics committee waived the requirement for informed consent. If patients provided informed written consent to have data from their medical records used in research, please include this information.

2. Please send a completed 'Competing Interests' statement, including any COIs declared by your co-authors. If you have no competing interests to declare, please state "The authors have declared that no competing interests exist". Otherwise please declare all competing interests beginning with the statement "I have read the journal's policy and the authors of this manuscript have the following competing interests:"

3. We noticed that you used "unpublished data" in the manuscript. We do not allow these references, as the PLOS data access policy requires that all data be either published with the manuscript or made available in a publicly accessible database. Please amend the supplementary material to include the referenced data or remove the references.

4. We do not publish any copyright or trademark symbols that usually accompany proprietary names, eg (R), (C), or TM  (e.g. next to drug or reagent names). Please remove all instances of trademark/copyright symbols throughout the text, including OCAP®.

5. For studies involving third-party data, we encourage authors to share any data specific to their analyses that they can legally distribute. PLOS recognizes, however, that authors may be using third-party data they do not have the rights to share. When third-party data cannot be publicly shared, authors must provide all information necessary for interested researchers to apply to gain access to the data. (https://journals.plos.org/plosone/s/data-availability#loc-acceptable-data-access-restrictions)

For any third-party data that the authors cannot legally distribute, they should include the following information in their Data Availability Statement upon submission:

1) A description of the data set and the third-party source

2) If applicable, verification of permission to use the data set

3) Confirmation of whether the authors received any special privileges in accessing the data that other researchers would not have

4) All necessary contact information others would need to apply to gain access to the data

If the reviewer comments include a recommendation to cite specific previously published works, please review and evaluate these publications to determine whether they are relevant and should be cited. There is no requirement to cite these works unless the editor has indicated otherwise.

**Additional Editor Comments (if provided):**

[Note: HTML markup is below. Please do not edit.]

Reviewers' comments:

Reviewer's Responses to Questions

**Comments to the Author**

1. Does this manuscript meet PLOS Global Public Health’s publication criteria?

Reviewer #1: Yes

**********

2. Has the statistical analysis been performed appropriately and rigorously?

Reviewer #1: Yes

**********

3. Have the authors made all data underlying the findings in their manuscript fully available (please refer to the Data Availability Statement at the start of the manuscript PDF file)?

The PLOS Data policy

Reviewer #1: Yes

**********

4. Is the manuscript presented in an intelligible fashion and written in standard English?

Reviewer #1: Yes

**********

**Reviewer #1:**  This study reports original research.

Results have not been reported elsewhere.

Methodology was good and statistical analysis used appropriate methods that were clearly described.

The paper claims that vitamin C deficiency is highly prevalent in the Indigenous people of Northern Saskatchewan. This high prevalence is attributed to structural inequalities and socioeconomic barriers disrupting food systems.

This claim is significant as it counters the prevailing assumption in high-income countries that vitamin C deficiency and scurvy are rare, of mainly historical interest.

The authors have placed the condition of vitamin C deficiency in the historical context of the European exploration, settlement and colonisation of Canada. They have described the disruption of traditional Indigenous food systems by modern commercial ultra-processed foods and the overall poverty and food insecurity of the local First Nations population. However, they have not referred to more recent literature describing the presence of vitamin C deficiency in clinical populations in high-income countries with different histories, or referred to the epidemiology of vitamin C deficiency in non-Indigenous populations in these countries.

The data presented do support claims that vitamin C deficiency is more common than previously suspected amongst the Indigenous people of Northern Saskatchewan. However, the small convenience sample cannot provide an estimate of prevalence in the clinic population or general population. Nor is it clear how representative this sample is of the population using the clinic: the only demographic information presented about the population is First Nations status and low-income status, but there is no more detailed breakdown of age, gender or health conditions for the population as a whole or the clinic patients, which might serve to compare with the study sample to identify sources of bias.

It would strengthen the study to have a comparison between the study sample and the clinic patient population as a whole in respect of age, gender, First Nations status, income, geographical location (urban/regional/remote), as well as prevalence of chronic disease (eg diabetes) and presenting conditions.

It is not clear what proportion of clinic patients were tested for vitamin C deficiency.

The time period of data collection includes 50 tests ordered between August 2023 and March 2024, plus one ordered between January 2011 and July 2023. It would seem reasonable to exclude the sole test ordered before August 2023 and include only those ordered in the more recent 8 month time period, which would be more relevant to contemporary practice.

The study is strong in presenting the evidence that vitamin C deficiency is common in clinical practice at this health service: with 37 cases of low/deficient status identified in 8 months (ie one or two cases per week). However the authors’ claims regarding causation are assertions based on common sense, rather than hypotheses generated from the data. There are theory-based arguments that could be made about the causal chain leading to vitamin C deficiency amongst Indigenous people, but these are not presented.

The recommendations made are reasonable, but again are based on common sense rather than scientific evidence. It would be reasonable to call for further research that might establish population prevalence and high-risk sub-populations, which would enable a cost-benefit analysis for potential interventions.

The study protocol is clearly presented and seems to have been followed by the researchers.

The study methods are standard and do not warrant publication in their own right.

The manuscript should be considered for publication after some modification: (a) the inclusion of more detailed data about the population from which the sample was drawn, enabling identification of demographic or clinical differences that might have biased the analysis; (b) the reference to recent evidence about the epidemiology of vitamin C deficiency in high-income countries.

Original data are held in accordance with data sovereignty principles.

The study conforms to relevant guidelines such as appropriate for statistical analysis.

The methodology is able to be used by other researchers to reproduce results.

No software was created by the authors.

The manuscript is clearly written and generally intelligible, but would benefit from inclusion of a table comparing participants with non-classical manifestations of vitamin C deficiency to those with classical symptoms. Given this is mainly a descriptive study, it would be beneficial to show clinicians the demographic and clinical characteristics of patients with vitamin C deficiency.

**********

what does this mean?). If published, this will include your full peer review and any attached files.

**Do you want your identity to be public for this peer review?**  If you choose “no”, your identity will remain anonymous but your review may still be made public.

For information about this choice, including consent withdrawal, please see our Privacy Policy

Reviewer #1: **Yes:**  Christopher Lemoh

**********

[NOTE: If reviewer comments were submitted as an attachment file, they will be attached to this email and accessible via the submission site. Please log into your account, locate the manuscript record, and check for the action link "View Attachments". If this link does not appear, there are no attachment files.]

# Author comment: Author response to Decision Letter 1

**DOI:** 10.1371/journal.pgph.0007464.r002

30 Apr 2026

Attachment Submitted filename: response to reviewers_PLOS GPH_vit C.pdf

# Peer review: Decision Letter 1

**DOI:** 10.1371/journal.pgph.0007464.r003

**Authors:** Martin Mickelsson

17 Jun 2026

PGPH-D-25-03694R1

Vitamin C Deficiency in a Northern Saskatchewan Population, Canada

PLOS Global Public Health

Dear Dr. Jeffrey David Connor Irvine,

Thank you for submitting your manuscript to PLOS Global Public Health. After careful consideration, we feel that it has merit but does not fully meet PLOS Global Public Health’s publication criteria as it currently stands. Therefore, we invite you to submit a revised version of the manuscript that addresses the points raised during the review process.

Please submit your revised manuscript by 16 July 2026. If you will need more time than this to complete your revisions, please reply to this message or contact the journal office at globalpubhealth@plos.org. When you're ready to submit your revision, log on to https://www.editorialmanager.com/pgph/ and select the 'Submissions Needing Revision' folder to locate your manuscript file.

Please include the following items when submitting your revised manuscript:

- A letter that responds to each point raised by the editor and reviewer(s). You should upload this letter as a separate file labeled 'Response to Reviewers'.
- A marked-up copy of your manuscript that highlights changes made to the original version. You should upload this as a separate file labeled 'Revised Manuscript with Track Changes'.
- An unmarked version of your revised paper without tracked changes. You should upload this as a separate file labeled 'Manuscript'.

Guidelines for resubmitting your figure files are available below the reviewer comments at the end of this letter.

As the corresponding author, your ORCID iD is verified in the submission system and will appear in the published article. PLOS supports the use of ORCID, and we encourage all coauthors to register for an ORCID iD and use it as well. Please encourage your coauthors to verify their ORCID iD within the submission system before final acceptance, as unverified ORCID iDs will not appear in the published article. *Only*  the individual author can complete the verification step; PLOS staff *cannot*  verify ORCID iDs on behalf of authors.

We look forward to receiving your revised manuscript.

Kind regards,

Martin Mickelsson, Ph.D.

Academic Editor

PLOS Global Public Health

**Journal Requirements:**

If the reviewer comments include a recommendation to cite specific previously published works, please review and evaluate these publications to determine whether they are relevant and should be cited. There is no requirement to cite these works unless the editor has indicated otherwise.

Please review your reference list to ensure that it is complete and correct. If you have cited papers that have been retracted, please include the rationale for doing so in the manuscript text, or remove these references and replace them with relevant current references. Any changes to the reference list should be mentioned in the rebuttal letter that accompanies your revised manuscript. If you need to cite a retracted article, indicate the article’s retracted status in the References list and also include a citation and full reference for the retraction notice.

**Additional Editor Comments (if provided):**

[Note: HTML markup is below. Please do not edit.]

Reviewers' comments:

Reviewer's Responses to Questions

**Comments to the Author**

Reviewer #1: All comments have been addressed

Reviewer #2: (No Response)

**********

publication criteria?

Reviewer #1: Yes

Reviewer #2: Partly

**********

3. Has the statistical analysis been performed appropriately and rigorously?

Reviewer #1: Yes

Reviewer #2: I don't know

**********

4. Have the authors made all data underlying the findings in their manuscript fully available (please refer to the Data Availability Statement at the start of the manuscript PDF file)?

The PLOS Data policy

Reviewer #1: Yes

Reviewer #2: Yes

**********

5. Is the manuscript presented in an intelligible fashion and written in standard English?

Reviewer #1: Yes

Reviewer #2: Yes

**********

**Reviewer #1** : (No Response)

**Reviewer #2:**  • Line 182: If Indigenous Identity is not available why offer the percentage of 83.9%? Where did this percentage come from? Is it truly reasonable to assume that the clinic population directly mirrors the population in that area?

• Line 209: The part about smoking status should be in the methods rather than the results if you were unable to assess.

• Line 214: the wording did not reach statistical significance implies to some readers that it may have been close to significance when it was not. I would suggest the wording “this difference was not statistically significant.”

• Methods/Results: Where are the p-results for each paired t-test? I see you report some (e.g., Line 177) but it’s unclear what the p-values are for each paired t-test. I’d recommend reporting these, as it’s hard for the reader to interpret what differences were tested and what had significance based on the way it is currently written.

**********

what does this mean?). If published, this will include your full peer review and any attached files.

**Do you want your identity to be public for this peer review?**  If you choose “no”, your identity will remain anonymous but your review may still be made public.

For information about this choice, including consent withdrawal, please see our Privacy Policy

Reviewer #1: **Yes:**  Christopher Lemoh

Reviewer #2: No

**********

[NOTE: If reviewer comments were submitted as an attachment file, they will be attached to this email and accessible via the submission site. Please log into your account, locate the manuscript record, and check for the action link "View Attachments". If this link does not appear, there are no attachment files.]

**Figure Resubmissions:**

Attachment Submitted filename: Vit C Saskatchewan.docx

# Author comment: Author response to Decision Letter 2

**DOI:** 10.1371/journal.pgph.0007464.r004

19 Jun 2026

Attachment Submitted filename: Response to Reviewer 2.pdf

# Peer review: Decision Letter 2

**DOI:** 10.1371/journal.pgph.0007464.r005

**Authors:** Katrien Janin

31 Jul 2026

PGPH-D-25-03694R2

Vitamin C Deficiency in a Northern Saskatchewan Population, Canada

PLOS Global Public Health

Dear Dr. Irvine,

Thank you for submitting your manuscript to PLOS Global Public Health. After careful consideration, we feel that it has merit but does not fully meet PLOS Global Public Health’s publication criteria as it currently stands. Therefore, we invite you to submit a revised version of the manuscript that addresses the points raised during the review process.

Your manuscript has now been reassessed. While the reviewers acknowledge the improvements made to the manuscript, they have also highlighted a few remaining issues that should be addressed. Please take these points into careful consideration when preparing your revised manuscript and your point‑by‑point response.

Please submit your revised manuscript by Sep 28 2026 11:59PM. If you will need more time than this to complete your revisions, please reply to this message or contact the journal office at globalpubhealth@plos.org. When you're ready to submit your revision, log on to https://www.editorialmanager.com/pgph/ and select the 'Submissions Needing Revision' folder to locate your manuscript file.

Please include the following items when submitting your revised manuscript:

- A letter that responds to each point raised by the editor and reviewer(s). You should upload this letter as a separate file labeled 'Response to Reviewers'.
- A marked-up copy of your manuscript that highlights changes made to the original version. You should upload this as a separate file labeled 'Revised Manuscript with Track Changes'.
- An unmarked version of your revised paper without tracked changes. You should upload this as a separate file labeled 'Manuscript'.

Guidelines for resubmitting your figure files are available below the reviewer comments at the end of this letter.

As the corresponding author, your ORCID iD is verified in the submission system and will appear in the published article. PLOS supports the use of ORCID, and we encourage all coauthors to register for an ORCID iD and use it as well. Please encourage your coauthors to verify their ORCID iD within the submission system before final acceptance, as unverified ORCID iDs will not appear in the published article. *Only*  the individual author can complete the verification step; PLOS staff *cannot*  verify ORCID iDs on behalf of authors.

We look forward to receiving your revised manuscript.

Kind regards,

Katrien G. Janin, PhD

Staff Editor

PLOS Global Public Health

Journal Requirements:

If the reviewer comments include a recommendation to cite specific previously published works, please review and evaluate these publications to determine whether they are relevant and should be cited. There is no requirement to cite these works unless the editor has indicated otherwise.

Additional Editor Comments (if provided):

[Note: HTML markup is below. Please do not edit.]

Reviewers' comments:

Reviewer's Responses to Questions

**Comments to the Author**

Reviewer #1: All comments have been addressed

Reviewer #3: (No Response)

**********

publication criteria?

Reviewer #1: Yes

Reviewer #3: Partly

**********

3. Has the statistical analysis been performed appropriately and rigorously?

Reviewer #1: Yes

Reviewer #3: No

**********

4. Have the authors made all data underlying the findings in their manuscript fully available (please refer to the Data Availability Statement at the start of the manuscript PDF file)?

The PLOS Data policy

Reviewer #1: No

Reviewer #3: No

**********

5. Is the manuscript presented in an intelligible fashion and written in standard English?

Reviewer #1: Yes

Reviewer #3: No

**********

Reviewer #1: The revised manuscript addresses all issues noted in the peer review.

The only minor suggestion is regarding Line 73: "anecdotal reports from physicians..." where it might be helpful to specify which physicians reported cases o scurvy, i.e. "anecdotal reports from local physicians...".

Reviewer #3: The study highlights the re-emergence of scurvy and vitamin C deficiency among marginalized Indigenous populations in a high-income country. It is noted for its focus on health equity, adherence to OCAP principles, and relevant policy recommendations, However, several critical questions remain that must be addressed:

1-The use of a convenience sample based on clinical suspicion introduces significant selection bias. The 53% deficiency rate likely reflects a high-risk subgroup rather than the general population. This limitation must be explicitly stated in the Abstract and Results to avoid misleading readers.

2-Due to the small sample size (n=48), the authors should avoid over-interpreting P-values for age and sex. The study should be framed as 'exploratory' or 'hypothesis-generating' rather than definitive.

3-While the 31% deficiency rate among asymptomatic patients is clinically significant, the authors should discuss whether 'non-classical' symptoms (e.g., fatigue or joint pain) could serve as more sensitive screening triggers for this population.

4-Given the stark contrast between the clinic population ($n=7,709$) and the tested group ($n=50$), the authors should explain the clinical criteria or specific triggers used to select this small fraction (0.64%) of patients in the Results section.

5-While the explanation of serum ascorbic acid testing challenges is excellent, the section is overly long. I suggest streamlining this part or moving the technical details to 'Supplementary Material' to improve the Discussion's flow.

6-Although the English is high quality, some paragraphs in the Discussion are overly complex. A final polish for readability is recommended to better engage a broader global public health audience.

**********

what does this mean?). If published, this will include your full peer review and any attached files.

**Do you want your identity to be public for this peer review?**  If you choose “no”, your identity will remain anonymous but your review may still be made public.

For information about this choice, including consent withdrawal, please see our Privacy Policy

Reviewer #1: **Yes:**  Christopher N Lemoh

Reviewer #3: No

**********

[NOTE: If reviewer comments were submitted as an attachment file, they will be attached to this email and accessible via the submission site. Please log into your account, locate the manuscript record, and check for the action link "View Attachments". If this link does not appear, there are no attachment files.]

# Author comment: Author response to Decision Letter 3

**DOI:** 10.1371/journal.pgph.0007464.r006

4 Sep 2026

Attachment Submitted filename: Response to Reviewers.docx

# Peer review: Decision Letter 3

**DOI:** 10.1371/journal.pgph.0007464.r007

**Authors:** Julia Robinson

27 Sep 2026

Vitamin C Deficiency in a Northern Saskatchewan Population, Canada

PGPH-D-25-03694R3

Dear Dr Irvine,

We are pleased to inform you that your manuscript 'Vitamin C Deficiency in a Northern Saskatchewan Population, Canada' has been provisionally accepted for publication in PLOS Global Public Health.

Before your manuscript can be formally accepted you will need to complete some formatting changes, which you will receive in a follow up email. A member of our team will be in touch with a set of requests.

Please note that your manuscript will not be scheduled for publication until you have made the required changes, so a swift response is appreciated.

IMPORTANT: The editorial review process is now complete. PLOS will only permit corrections to spelling, formatting or significant scientific errors from this point onwards. Requests for major changes, or any which affect the scientific understanding of your work, will cause delays to the publication date of your manuscript.

If your institution or institutions have a press office, please notify them about your upcoming paper to help maximize its impact. If they'll be preparing press materials, please inform our press team as soon as possible -- no later than 48 hours after receiving the formal acceptance. Your manuscript will remain under strict press embargo until 2 pm Eastern Time on the date of publication. For more information, please contact globalpubhealth@plos.org.

Thank you again for supporting Open Access publishing; we are looking forward to publishing your work in PLOS Global Public Health.

Best regards,

Julia Robinson

Executive Editor

PLOS Global Public Health

***********************************************************

Reviewer Comments (if any, and for reference):

Reviewer's Responses to Questions

**Comments to the Author**

Reviewer #1: All comments have been addressed

**********

publication criteria?

Reviewer #1: Yes

**********

3. Has the statistical analysis been performed appropriately and rigorously?

Reviewer #1: Yes

**********

4. Have the authors made all data underlying the findings in their manuscript fully available (please refer to the Data Availability Statement at the start of the manuscript PDF file)?

The PLOS Data policy

Reviewer #1: No

**********

5. Is the manuscript presented in an intelligible fashion and written in standard English?

Reviewer #1: Yes

**********

Reviewer #1: All comments have been addressed.

The restrictions on data availability are noted and arre appropriate.

**********

what does this mean?). If published, this will include your full peer review and any attached files.

**Do you want your identity to be public for this peer review?**  If you choose “no”, your identity will remain anonymous but your review may still be made public.

For information about this choice, including consent withdrawal, please see our Privacy Policy

Reviewer #1: **Yes:**  Christopher Lemoh

**********
